# S100 Proteins in Pancreatic Cancer: Current Knowledge and Future Perspectives

**DOI:** 10.3389/fonc.2021.711180

**Published:** 2021-08-30

**Authors:** Yu Wu, Qi Zhou, Fangyue Guo, Mingming Chen, Xufeng Tao, Deshi Dong

**Affiliations:** ^1^Department of Clinical Pharmacy, First Affiliated Hospital of Dalian Medical University, Dalian, China; ^2^College of Pharmacy, Dalian Medical University, Dalian, China; ^3^Laboratory of Integrative Medicine, First Affiliated Hospital of Dalian Medical University, Dalian, China; ^4^Institute (College) of Integrative Medicine, Dalian Medical University, Dalian, China; ^5^School of Chemical Engineering, Dalian University of Technology, Dalian, China

**Keywords:** S100 proteins, pancreatic cancer, biomarkers, mechanisms, therapeutic targets

## Abstract

Pancreatic cancer (PC) is a highly malignant tumor occurring in the digestive system. Currently, there is a lack of specific and effective interventions for PC; thus, further exploration regarding the pathogenesis of this malignancy is warranted. The S100 protein family, a collection of calcium-binding proteins expressed only in vertebrates, comprises 25 members with high sequence and structural similarity. Dysregulated expression of S100 proteins is a biomarker of cancer progression and prognosis. Functionally, these proteins are associated with the regulation of multiple cellular processes, including proliferation, apoptosis, growth, differentiation, enzyme activation, migration/invasion, Ca2+ homeostasis, and energy metabolism. This review highlights the significance of the S100 family in the diagnosis and prognosis of PC and its vital functions in tumor cell metastasis, invasion and proliferation. A further understanding of S100 proteins will provide potential therapeutic targets for preventing or treating PC.

## Introduction

Members of the S100 protein family were first discovered in the brains of certain species by Moore in 1965 (1). This family is a group of calcium-binding proteins expressed only in vertebrates (2, 3), including 25 known members in humans. The genes of 21 S100 family members (repetin, trichohyalin, filaggrin, and S100A1-S100A18) map to chromosome 1q21, while others share chromosomes 5q14 (S100Z), 4p16 (S100P), Xp22 (S100G), and 21q22 (S100B) (4–6). All S100 proteins have a certain degree of sequence and structural similarity, but each protein is encoded by a specific gene that has distinct differences in protein-expressing cells and biological functions (7, 8). Some members of the S100 protein family play an important role in the pathophysiology of certain cancer types (2, 9–12).

Pancreatic cancer (PC) is a common malignant tumor of the pancreas, and its early clinical symptoms are relatively insidious (13–15). Most patients are diagnosed at an advanced stage, usually accompanied by tumor cells spreading beyond the pancreas, and the prognosis is poor (15, 16). Data provided from 2009 to 2015 by the American Cancer Society estimate that the total survival rate of PC patients is only 9%, and the number of deaths from PC ranks 4th among malignancies (17). PC is expected to become the second leading cause of cancer-related deaths by 2030 (13). There is currently a lack of clinically effective cures, and surgical resection may be the only chance to cure PC (15, 18). Recently, advances in adjuvant chemotherapy have led to a relative improvement in the prognosis of PC patients; however, the 5-year survival rate is only 20% for patients undergoing complete resection, chemotherapy, and radiation (19–21). Thus, it is of great importance to understand PC onset, which will contribute to early diagnosis and improve prognosis.

Many studies have shown that certain members of the S100 family are associated with PC (22–24). Although several excellent reviews on the partial association of the S100 protein family with PC have been published (2, 25, 26), here, we try to fully focus on the role of certain members of the S100 protein family in PC based on the latest studies. In this review, we critically study the role of the S100 protein family in PC diagnosis or treatment and the contribution of S100 signaling to the biology of PC-related cells, providing a relatively comprehensive reference for the S100 protein family as a target for prevention or treatment of PC.

## S100 Protein Family Links to PC Diagnosis and Prognosis

Although some progress has been made in imaging techniques and tumor markers, early diagnosis of PC is still challenged by the absence of precise biomarkers. At present, serum carbohydrate antigen 19-9 (CA19-9) is the sole diagnostic marker in PC authorized by the U.S. Food and Drug Administration, but it is also sensitive to the host’s inflammatory response and obstructive jaundice; thus, it is not specific for PC (27). Therefore, it is necessary to develop new biomarkers for early PC diagnosis, patient selection for optimal management, and prognosis determination. To date, with the gradual deepening understanding of S100 proteins as diagnostic, treatment-predictive and prognostic biomarkers in PC, physicians are interested in the S100 protein family.

### S100A2: A Biomarker of PC Progression or Negative Prognosis

There is controversy about the actions of S100A2 in tumor development. It is documented to be a candidate tumor suppressor and has also been found to be a promoter of certain cancer types (28–30). The pathological process of PC progresses from pancreatic ductal epithelial hyperplasia to dysplasia to carcinoma *in situ* to invasive carcinoma (31, 32). PC has 3 typical precursor lesions, including mucinous cystic neoplasm (MCN), intraductal papillary mucinous neoplasm (IPMN), and pancreatic intraepithelial neoplasia (PanIN) (33–35). S100A2 expression in PanIN and invasive ductal carcinoma (IDC) cells is higher than that in normal ductal cells, pancreatitis-affected epithelial cells (PAEs), and IPMNs and is upregulated in IDC cells from poorly differentiated adenocarcinoma compared to IDC cells from normally differentiated adenocarcinoma (36). S100A2 is commonly considered to be a sign of PC progression due to its significance in differentiation of adenocarcinoma (36, 37). Pancreatic ductal adenocarcinoma (PDAC) accounts for more than 90% of all pancreatic malignancies (38) and can be divided into 4 molecular subtypes: pancreatic progenitor, immunogenic, aberrantly differentiated endocrine exocrine (ADEX), and squamous (39). The hypomethylation and increased expression of S100A2 determine the prognosis of the “squamous” (also known as QM or basal) subtype driven by TP53 and KDM6A mutations (39–41). Meanwhile, studies have linked abnormal expression of S100A2 protein to the poor survival rate of PDAC patients (42). High S100A2 expression has been observed in the metastatic site but not in the primary tumor, suggesting that it may be a marker of tumor metastasis (42). Moreover, S100A2 is a good resectable marker of PC, and measuring its expression in biopsy samples has potential clinical application value (42, 43). Samples from patients with survival <1000 days after PC resection express high levels of S100A2 compared with those from patients who survived >1000 days. Even after surgery, S100A2 functionally promotes the expression of tumorigenesis and metastasis molecules, whose high expression is an independent marker of poor prognosis (36, 42, 44). Ohuchida et al. predicted that S100A2 expression is correlated with longer overall survival (OS) and disease-free survival (DFS) in patients treated with adjuvant therapy (37). Overall, the current research suggests that S100A2 overexpression is a biomarker of tumor progression or negative prognosis in PC patients (36).

### S100A4: A Risk Factor for PC

S100A4, also known as Mt1 or Fsp1, is mostly not expressed in normal tissues but is highly expressed in various tumors. In particular, the S100A4 level in PC cells is higher than in nonmalignant tumors or nontumor epithelial cells, and S100A4 is carcinogenic in PC (45–47). S100A4 expression is positively related to the tumor-node-metastasis (TNM) staging and tumor size in PC; the larger the tumor size or the higher the TNM stage, the higher S100A4 expression is (48). S100A4 may be a key regulator in liver metastasis of PC (49) and a potential marker of lymph node metastasis, but there is no obvious correlation between S100A4 and histological type or distant metastasis status (50). In addition, Tsukamoto et al. emphasized the important role of S100A4 in the invasiveness of PC, particularly with perineural invasion and invasion patterns (51). A meta-analysis of 474 patients with PC indicated that S100A4 is a potential adverse factor in PC prognosis, whose positive expression is considerably related to a lower 3-year OS rate (50), suggesting that S100A4 may be a potential indicator to predict the survival rate of patients (44, 48). Lee et al. performed immunohistochemical analysis of epithelial-to-mesenchymal transition (EMT)- and metastasis-related proteins in PDAC and confirmed that the expression of CD24 and S100A4 are important independent predictors of early recurrence and a low survival rate of PDAC patients (52). In addition, S100A4 knockdown increased the sensitivity of a PDAC cell line to gemcitabine (the first-line drug for advanced PC) treatment (53), thereby improving the therapeutic effect of the drug or the survival time of the patient. Radiation therapy is also an important strategy for treating PC patients. The expression level of S100A4 increases with continuous radiation and is positively related to the radiation resistance of PC cells (47). Whole-tumor evaluation with magnetic resonance imaging (MRI) and texture analysis have established a model that predicts S100A4 overexpression as an imaging biomarker of PDAC (54). S100A4 can also be combined with other tumor biomarkers for early PC diagnosis and determination of PC prognosis (55–59), and the combinations can improve the accuracy of distinguishing PC from normal tissues to varying degrees. In conclusion, high S100A4 expression is not only a sign of pancreatic tumor malignancy but also a potential marker of PC metastasis and poor prognosis.

### S100A6: A Biomarker for PC Lesions

Higher S100A6 mRNA expression levels were discovered in PC tissues than in noncancerous tissues (60–63), and S100A6 expression was mainly restricted to the nuclei in PC cells (64). A significant difference in S100A6 expression has also been detected in pancreatic juice between PC patients and non-PC patients, and the survival time of PC patients with high nuclear S100A6 is shortened (62, 64). The expression of S100A6 gradually increases in the process of PC carcinogenesis and may be a biomarker of high-risk lesions in PC (65). Compared with pancreatitis-affected epithelial or normal cells, S100A6 is overexpressed in IDC and IPMN cells, supporting the idea that measurement of S100A6 might help distinguish PDAC or IPMN from CP (60, 65). The upregulation of S100A6 may be an early event in PC; therefore, detecting the S100A6 mRNA level may be a promising tool for diagnosing PC, which will benefit early diagnosis and increase the chance of cure (64). EUS-FNA is a relatively accurate technique for assessment and staging of PC; however, it also has uncertainties in up to 20% of cases (66). The S100A6 mRNA levels in EUS-FNA samples quantified by real-time PCR have higher specificity and sensitivity for PDAC diagnosis, which can reduce false-negative diagnoses *via* EUS-FNA cytology and improve the accuracy of decision-making (67).

### S100A8/S100A9: Potential Promoters of PC Development

S100A8 and S100A9 are overexpressed in acute pancreatitis (AP), CP, and PC tissues (68, 69), but they are rarely expressed in CP tissues with severe fibrosis, normal pancreatic tissues, and ductal cells (69, 70). CP is a well-known independent risk factor for PC (71–73).The absence of S100A9 can alleviate AP, thereby reducing the risk of recurrent AP evolving into CP (74). The poor prognosis of PDAC patients is related to the high expression of S100A8 or S100A9 in pancreatic duct fluid. S100A8 or S100A9 may be potential prognostic biomarkers of PC (75). Samonig et al. analyzed more than 400 proteins in stem cell-like pancreatic tumor-initiating cells (TICs) and nontumor-initiating cells (non-TICs) through differential proteomics (PTX) and then nominated S100A8, S100A9, and galactin-3-binding protein LGALS3BP (MAC-2-BP) as putative driver genes for pancreatic TICs, but this conclusion needs further verification (76). In summary, the expression of S100A8 or S100A9 has a certain indirect effect on PC occurrence and development and has a certain positive effect on PC prevention and treatment.

### S100A11: A Two-Way Regulatory Factor for PC

S100A11 expression is upregulated in PC (77–79), breast cancer (80), nonsmall cell lung cancer (81), and colorectal cancer (82) but is decreased in bladder cancer (83). Immunohistochemical analysis of 78 pairs of human PC tissues and adjacent nontumor tissue specimens revealed that the expression of S100A11 in PC tissues is considerably higher than that in surrounding nontumor tissues and that S100A11 is mainly distributed in the cytoplasm of PC cells. Further research found that S100A11 expression increased in the early stages of PC, and multivariate analysis indicated that S100A11 was an independent adverse prognostic factor in PC (78). In contrast, S100A11 expression is downregulated as cancer progresses to a worse phenotype. These seemingly contradictory results support the notion that elevated S100A11 is beneficial only for early diagnosis of PC; however, S100A11 can also be used as a tumor suppressor gene in PC development (77). Thus, analyzing the level of S100A11 in pancreatic juice may be a feasible way to diagnose PC or high-risk PC lesions. Although there was no significant correlation between S100A11 expression and tumor location, TNM stage, tumor diameter, distant metastasis, or the level of CA19-9, S100A11 expression was correlated with lymph node metastasis and tissue differentiation in PC (78).

### S100P: An Indicator of Early PC Occurrence

S100P was originally identified in the human placenta. Using cDNA array, serial analysis of gene expression (SAGE), and tissue array data to compare the overall gene expression profile of PC with that of normal pancreatic tissues, Crnogorac-Jurcevic et al. found that a specific increase in S100P expression occurs only in the tumor epithelium of PC, indicating that S100P may be a promising biomarker to monitor PC (61). S100P can be used as a biomarker of the early development of PC; its expression level in PC and IPMN is significantly higher than in nontumor pancreatic tissues, and its expression level gradually increases with the grade of PanIN (60, 84–87). Compared with patients with pancreatitis, the content of S100P in the pancreatic juice of PC and IPMN patients was significantly increased. Thus, measuring the expression level of S100P in pancreatic juice might help distinguish pancreatitis from tumor disease for early screening and diagnosis of PC (85). Since S100P is a specific and sensitive marker, it is possible to detect the concentration of S100P in duodenal fluid (DF) based on upper gastrointestinal endoscopy (GIE) or endoscopic ultrasonography, which may be helpful for early PDAC screening (88). In addition, evaluation of S100P expression can be combined with EUS-FNA quantitative analysis to improve the accuracy of diagnosis of PC and nontumor lesions (89–91) or be applied in fine-needle aspiration biopsy (FNAB) specimens prepared in cell blocks and smears to test for PDAC (92, 93).

### Others

A few studies have reported a relationship between other S100 proteins and PC. S100A7, also known as psoriasin, commonly exists in the nucleus or cytoplasm in various cells and can be secreted under certain circumstances. S100A7 has a high level of expression in locally advanced and invasive PC but low expression in distant metastatic primary PC. Interestingly, there was no significant difference in S100A7 expression between PC and adjacent tissues (94). S100A10 is regarded as a new biomarker of PDAC, and its expression in normal pancreatic ducts and PanIN-1A is markedly lower than that in PanIN-1B, PanIN-2, PanIN-3, and PDAC (95, 96). S100A14 is overexpressed in human PDAC cell lines and tissues, and its expression level is positively correlated with advanced cancer stages and negatively correlated with the survival time of PDAC patients (97, 98). A recent study demonstrated that S100A14 shows a certain degree of carcinogenicity and may enhance gemcitabine resistance, promoting PDAC progression, and suppression of its expression can inhibit PDAC formation (97). S100A16 is distinctly upregulated in clinical PDAC samples but unchanged/downregulated in nontumor pancreatic tissues. S100A16 expression is negatively related to the OS and RFS of patients with PC by promoting the growth, proliferation, metastasis, and invasion of PDAC cells *in vivo* and *in vitro* (99, 100). All of these proteins are expected to be potential markers of PC; however, their functions in the course of PC have only slowly begun to be revealed in recent years, and further research is needed.

In conclusion, some members of the S100 protein family play an important role in the development and treatment of poorly curable PC (24, 37, 101). Although their specific role is not yet clear, a large number of studies have provided evidence for regulation of S100 proteins and their involvement in the pathophysiological process of PC. At present, S100A2, S100A4, S100A6, S100A8/S100A9, S100A11 and S100P seem to be the S100 family proteins most related to PC, showing certain potential to regulate or predict the occurrence, development or prognosis of PC. It is believed that further studies on S100 proteins in the future will improve the clinical treatment of PC to varying degrees.

## S100 Family-Mediated Cellular Signaling Network in PC

**Figure 1:**
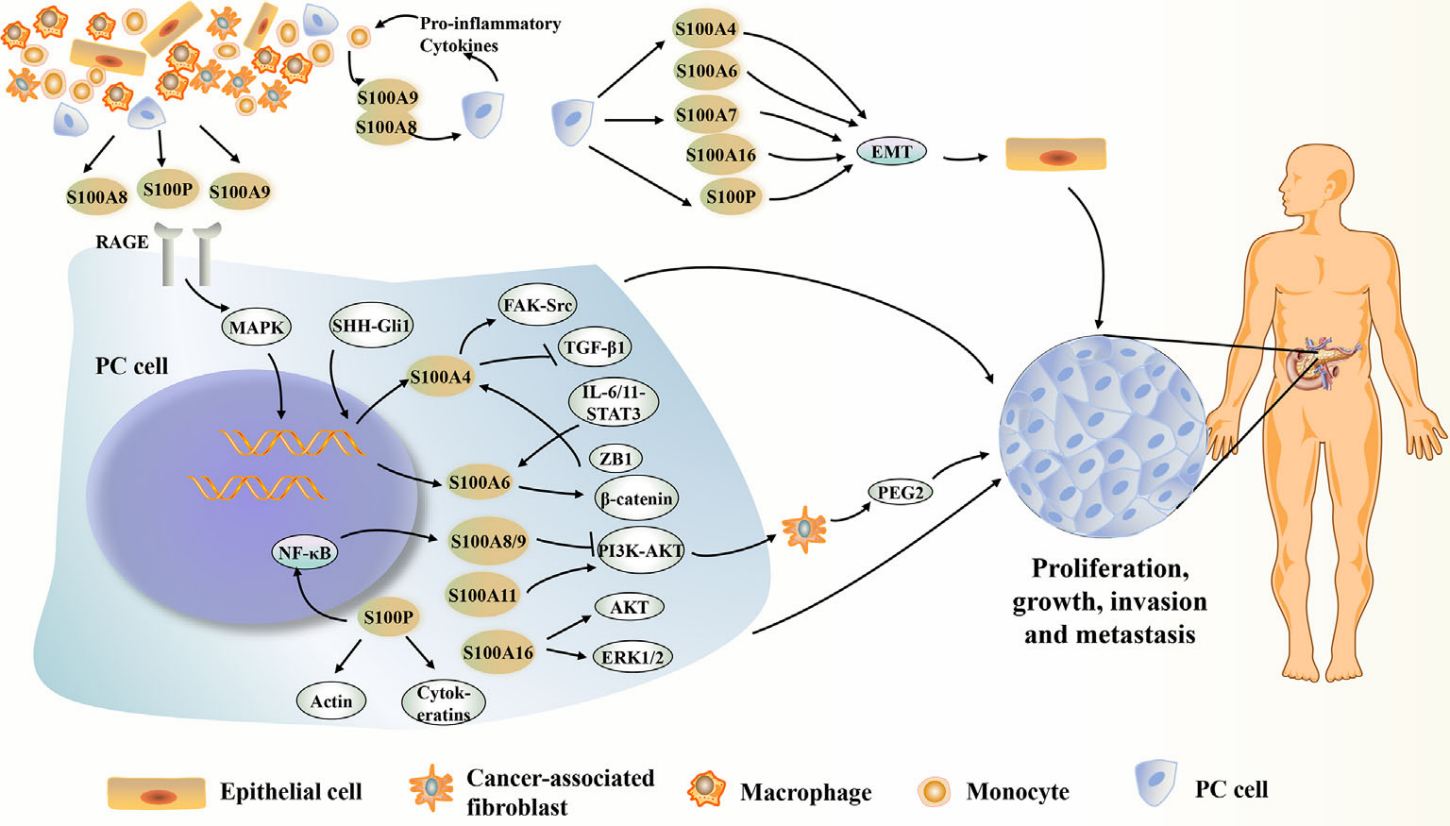
Summary of the potential mechanisms of several S100 proteins affecting PC. S100 proteins present in or derived from PC cells and surrounding stromal cells can play an important intracellular and extracellular role in the PC development. S100A4, S100A6, S100A7, S100A8, S100A9, S100A10, S00A11, S10016 and S100P show increased expression during PC development. They can act on certain proteins or signaling pathways inside or outside the cell, directly or indirectly affecting the growth, proliferation, metastasis or invasion of PC. However, S00A14 can inhibit PC metastasis or apoptosis of PC cells. EMT, epithelial-mesenchymal transition; ERK1/2, extracellular signal-regulated kinase 1/2; FAK, focal adhesion kinase; IL-6/11, interleukin-6/11; MAPK, mitogen-activated protein kinase; NF-κB, nuclear factor kappa-light-chain-enhancer of activated B cells; PEG2, prostaglandin E2; PI3K/AKT, phosphatidylinositol-3-kinase (PI3K)/protein kinase B (AKT); RAGE, advanced glycosylation end-product receptor; STAT3, signal transducer and activator of transcription 3; TGF-β1, transforming growth factor beta-1.

S100 proteins are binding proteins involved in intracellular Ca2+ homeostasis, and play a role in proliferation, growth, differentiation, apoptosis, enzyme activation, migration/invasion, and regulation of energy metabolism in both a Ca2+-dependent and non-Ca2+-dependent manner (8). Secreted S100 proteins interact with a variety of cell surface receptors, such as receptor for advanced glycation end products (RAGE), Toll-like receptor 4 (TLR4), G-protein-coupled receptors, heparin sulfate proteoglycan, and scavenging receptor, acting through autocrine and paracrine pathways (24, 79, 102–104). Recently, numerous studies have revealed the regulatory mechanisms of S100 family members in the complex biosignaling network in PC (**Figure 1**).

### S100A2

Currently, the biological activity of S100A2 in tumors has not yet been elucidated, and thus, its function in PC is still a mystery. Gene mutations in PDAC often include mutations in the oncogene KRAS and inactivation of the tumor suppressor genes TP53, SMAD4 and CDKN2A (105–108). Wild-type TP53 induces increased transcription of S100A2; in turn, S100A2 activates the transcriptional activity of TP53 (109–111). However, the complex interaction between increased S100A2 and TP53 in PC is not yet clear. A small molecular affinity between S100A2 and RAGE has been demonstrated in a strictly calcium-dependent manner. Further exploration to understand whether S100A2 binding to RAGE is involved in PC pathology is necessary (112).

### S100A4

The overexpression of S100 protein in PC is related to hypomethylation of S100A4 gene (45, 113). Using an orthotopic human PC xenograft mouse model, S100A4 has been concluded to accelerate PC progression by promoting cancer cell growth, survival, invasion, migration, and angiogenesis *in vivo* (22). S100A4 expression in PC cells can avoid transforming growth factor beta (TGF-β)-induced growth inhibition and apoptosis of PC cells, and can promote the survival, proliferation, and migration of PC cells through the Src-focal adhesion kinase (FAK)- mediated dual signaling pathway (22). Additionally, intracellular S100A4 is positively correlated with matrix metalloproteinases (MMPs), such as MMP-2 and MMP-9, and negatively correlated with E-cadherin to promote PC cell metastasis and invasion (114–116). Takahiro Tabata et al. used the RNA interference (RNAi) method to specifically knock down the expression of S100A4 in human PC cell lines *in vitro*, which induced G2 arrest and apoptosis and reduced cell migration. In addition, microarray analysis showed that knockdown of S100A4 can induce the expression of the tumor suppressor genes positive regulatory domain zinc finger protein 2 (PRDM2) and vasohibin-1 (VASH1) (117). Bcl-2 interacting protein 3 (BNIP3) is a member of the BH3-only subfamily of the Bcl-2 protein family, which induces apoptosis *via* the mitochondrial-dependent pathway under hypoxic conditions. BNIP3 has the biological activity of inducing PC cell apoptosis and increasing the sensitivity of tumor cells to gemcitabine; unfortunately, its expression is significantly downregulated in PC. Therefore, reactivation of BNIP3 may be an important therapeutic target for PC (118–122). Mahon et al. found that the S100A2 and S100A4 proteins were negatively correlated with BNIP3 expression profiles *in vitro* (53). S100A4 may be a key factor that promotes the EMT process in PC. Studies have also found that the Shh-Gli1 signaling pathway mediates the transcription of the target gene S100A4 in PC cells to regulate EMT and promote PC metastasis (123, 124). In PDAC cell lines, interleukin (IL)-6/11-STAT3 and zinc finger E-box binding homeobox 1 (ZEB1) synergistically upregulate S100A4/A6, thereby promoting PC cells invasion and EMT (24). However, the exact effects of S100A4 in PC remain unclear, and the current results regarding S100A4 in PC require further verification.

### S100A6

Inhibition of S100A6 reduces the invasiveness and proliferation of PC cells, and the possible mechanisms are that S100A6 activity may directly or indirectly regulate tumor proliferation/invasion/metastasis-related genes (62, 86). EMT is an important step in cancer invasion and metastasis. Downregulation of epithelial cadherin is a hallmark of EMT that occurs during various processes, including early tumor metastasis. Chen et al. demonstrated that S100A6 induces EMT and promotes cell migration and invasion in a β-catenin-dependent manner in human pancreatic cancer Panc-1 cells *in vitro* (125). In addition, S100A6 increases the expression of vimentin, β-catenin, and N-cadherin in Panc-1 cells while decreasing E-cadherin expression (125). Annexin A2 is a calcium-dependent protein that can promote PC development and metastasis, and it may also be a target for treatment of PC (126, 127). Both annexin 2 and S100A6 are expressed in the early stage of PC and are overexpressed with high frequency in invasive cancer. The combination of S100A6 and annexin 2 in PC cells can promote PC cell motility (128). Given that it is responsible for tumor occurrence and metastasis, S100A6 is a promising therapeutic target to treat PC. In view of the current research explaining the specific role of S100A6 in PC, there is still a long way to go.

### S100A8/A9

Immune system imbalance is one of the crucial facilitators of PDAC development. S100A8 and S100A9 proteins are overexpressed in PC (68, 69), and their complex may be one of the possible mediators of inflammation occurring in PDAC immunosuppression. Cytotoxic T-lymphocyte antigen 4 (CTLA4) is involved in regulating the immunosuppressive activity of T cells and plays a key role in the negative regulation of T cell activation (129). However, the S100A8 and S100A9 complexes can reduce CTLA4 expression on the surface of highly immunosuppressive CD33^+^CD14^+^HLA-DR^−^ monocytic myeloid-derived suppressive cells (MDSCs) *in vitro*, which is also a feature of the immunosuppressive phenotype (130, 131). RAGE protein is expressed in a variety of cells such as endothelial cells, tumor cells, macrophages, neutrophils, and mast cells (132, 133). S100A8/A9 secreted by monocytes can induce pancreatic tumor cells to secrete proinflammatory cytokines, including IL-8, fibroblast growth factor (FGF), and tumor necrosis factor-alpha (TNF-α), mediated in part by RAGE in pancreatic tumor cells. In turn, these cytokines induce S100A8 and S100A9 in monocytes to form a paracrine feedback loop, which may affect the invasion and migration of PC (134). RAGE is also involved in S100A8/A9-mediated activation of mitogen-activated protein kinase (MAPK) and nuclear factor kappa-light-chain-enhancer of activated B cells (NF-κB) signaling in PC progression, thereby promoting tumor growth, spread, and metastasis (134). Therefore, targeting S100A8/A9 is expected to become an effective treatment for inhibiting the progression of PC. Furthermore, Smad4 is widely expressed in normal pancreatic tissues and mesenchymal fibroblasts, and the expression of SMAD4 protein in PC tissues is significantly lower than that of adjacent tissues and normal pancreas (135). The Smad4 gene has a high frequency of mutations in PC, and the expression of S100A8 is associated with the tumor suppressor protein Smad4 in PC cells (69, 136, 137). Loss of Smad4 in the tumor microenvironment can change the state of PC in response to S100A8 (69, 137). S100A8/A9 inhibits the NF-κB and the phosphatidy-linositol-3-kinase (PI3K)/protein kinase B(AKT) pathways in PC cells in a Smad4-dependent manner, which may be a way to alleviate the course of PC (130, 138). To date, the specific effect of S100A8/A9 in PC is still unclear, and how protein complexes regulate the biological functions of PC cells is still a hot spot for future research. Furthermore, in view of the intracellular and extracellular activities of S100A8/A9 in pancreatitis, it will be important to determine whether and how S100A8/A9 is involved in pancreatitis-initiated PDAC.

### S100A11

Both S100A11 and TGF-β1 are significantly overexpressed in PC tissues, and S100A11 is correlated with TGF-β1/Smad4 signaling, but these proteins are independent of each other in regulating PANC-1 cell growth. Inhibition of TGF-β1 expression in the PANC-1 cell line *via* small interfering RNA (siRNA) downregulated the expression of the P21WAF1 tumor suppressor gene, blocked S100A11 from entering the nucleus from the cytoplasm, and promoted cell proliferation. However, silencing S100A11 can downregulate the expression of P21WAF1 in cells and promote cell apoptosis (139). It has also been clarified that S100A11 can upregulate the PI3K/AKT signaling pathway, thereby promoting the survival and proliferation of PANC-1 cells (23). These studies indicate that S100A11 may be a potential gene therapy target for PC. It is well known that the proliferation of PDAC-related fibroblasts accelerates PDAC progression. Extracellular S100A11 secreted by PDAC activates surrounding fibroblasts through the S100A11-RAGE-tumor progression locus 2 (TPL2)-cyclooxygenase 2 (COX2) pathway, which can induce the production of prostaglandin E2 (PGE2), a key soluble factor that accelerates PDAC cell motility and ultimately leads to an increase in the number of PDAC-derived circulating tumor cells (CTCs) (79). In addition, the extracellular S100A11 secreted by PDAC cells mediates the proliferation of neighboring fibroblasts through RAGE-MyD88-mTOR-p70 S6 kinase signaling, which in turn leads to fertilization of the PDAC interstitium and promotes PDAC growth (140). Although many studies have indicated that the regulation of cancer-associated fibroblasts (CAFs) through known targeted pathways may be a good choice to treat PDAC, the mechanism of S100A11 in fibroblasts is not yet clear.

### S100P

Using an oligonucleotide microarray-based method, the S100P genes have been found to be abnormally hypomethylated in PC. Further exploration revealed that S100P hypomethylation in PC is likely the cause of S100P mRNA overexpression (141). S100P directly stimulates tumor cell growth, movement, and invasion; protects PC cells from apoptosis or anoikis caused by chemotherapy drugs; or mediates changes in the cytoskeleton of carcinoma cells, thereby promoting growth, survival, and invasion of PC (86, 142). The upregulation of S100P in PDAC lymph node metastasis and its promotion of growth and invasion in PDAC make it a possible epithelial-specific target protein to treat primary or metastatic PC (143). S100P can activate RAGE, which may be an important factor causing the invasiveness of most PC lesions. Immunohistochemical analysis has been reported to confirm the expression of RAGE and its ligand S100P in human PDAC, and siRNA silencing of these genes can reduce the migration or growth of PDAC cells (142, 144). In mouse models, cromolyn, analog 5-methyl cromolyn (C5OH), and RAGE antagonist peptides block the binding of S100P to RAGE, thereby reducing the activity of NF-κB and inhibiting the growth and metastasis of PC (142, 145, 146). Meanwhile, S100P can activate the MAPK and NF-κB pathways in PC cell lines (145, 147, 148). Dakhel et al. reported that extracellular S100P stimulates BxPC3 cell line proliferation by inducing phosphorylation of IκBα and MMP-9 secretion and increases the survival rate of BxPC3 cell lines exposed to GEM. An anti-S100P monoclonal antibody can sabotage these activities and significantly delay liver metastasis and tumor growth (149). Therefore, a combination of S100P monoclonal antibody and targeted drugs or chemotherapy drugs may be a promising method to treat PC in the future. Barry et al. found that S100P promotes the transendothelial migration of PDAC cells in a zebrafish embryo model, indicating that S100P promotes infiltration/extravasation of cancer cells, which may be a key step in the blood spread of PC cells (150). Additionally, through *in vitro* experiments, studies have found that the spheroids of PC cells can cause the formation of circular chemorepellent-induced defects (CCIDs), thereby promoting lymphatic metastasis of cancer cells. Fortunately, S100P may be an effective target for inhibiting lymph node metastasis because these CCIDs in PC are partially regulated by S100P (101). Overexpression of S100P in Panc1 cells increases PC metastasis and invasion by inducing disruption of certain cytoskeletal proteins (including cytokeratin 8, 18, and 19), mediating disruption of the actin cytoskeletal network, altering the phosphorylation state of the actin regulatory protein cofilin and upregulating cathepsin D (86). These breakthrough discoveries are essential for improving the poor prognosis of PDAC patients, but the mechanisms are not yet fully understood.

### Others

Overexpression of S100A7 in PC cells increases the expression and activity of MMP-2 and MMP-9, leading to increased invasiveness of PC (94). It can also promote the aggregation and survival of PC cells that have lost their anchorage (94). Therefore, high expression of S100A7 is linked to growth, migration, and local invasion of PC, but the specific mechanisms underlying S100A7 actions in PC are largely unknown. S100A10 is overexpressed in PC, driven by specific promoter hypomethylation and KRAS. Knockout of S100A10 can reduce the surface plasminogen activation of PC cells and inhibit the invasiveness and growth of PC *in vivo* (96). Overexpression of S100A16 not only significantly promotes phosphorylated ATK (p-ATK) and phosphorylated extracellular signal-regulated kinase 1/2 (p-ERK1/2) in SW1990 and PANC-1 cells but also promotes invasion and proliferation of PDAC cells *via* AKT and ERK1/2 signaling in an FGF19-dependent manner. High expression of S100A16 inhibits the level of E-cadherin but increases the level of vimentin. In PDAC SW1990 cells, knocking out S100A16 can induce cell cycle arrest in G2/M phase and apoptosis, and in Aspc1 or PANC-1 cells, repression of S100A16 increases the percentage of annexin V+ cells and thus may also increase apoptosis (100). Twist1 is highly expressed in some malignant tumors, induces EMT, and enhances chemotherapeutic drug resistance (151–153). In one study, *in vitro* and *in vivo* experiments showed that activation of the signal transducer and activator of transcription 3 (STAT3) signaling pathway and upregulation of Twist1 expression mediate S100A16-induced EMT and promote metastasis of human PDAC cells (99). GATA binding protein 3 (GATA 3) is a transcription factor containing zinc finger domains and plays an important role in the occurrence and development of certain tumors (154–156). Immunohistochemistry has shown that PC cells have a strong and persistent cytoplasmic GATA-3 immune response (155, 157). The expression of GATA-3 is correlated with the mRNA expression of Smad-3, TGF-β receptors, and TGF-βs, and disruption of the transcriptional function of GATA-3 may enhance TGF-β signaling, and subsequently lead to malignant transformation of pancreatic cells, thus promoting PC development (157). The long noncoding RNA (lncRNA) GATA 3-antisense RNA1 (AS1)-microRNA (miR)-30b-5p-testis-expressed protein 10 (Tex10) axis has been found to regulate cell growth, apoptosis, invasion and apoptosis in PC tissues and cells, which may be related to the Wnt/β-catenin signaling pathway (158). The exact role of Gata-3 in PC cells needs further study to determine whether GATA-3 can be used as a biomarker or treatment target for PC.

Some studies have investigated inhibitors or existing drugs to inhibit S100 protein expression and the progression of PC. Ramatoulie Camara et al. used in silico methods to identify potential binding pockets in the NMR ensemble of S100P and successfully discovered several small molecule inhibitors that affect the S100P-RAGE interaction and S100P-mediated cell invasion, which can inhibit S100P-expressing PC cell invasion *in vitro*. This provides strong evidence of the potential of S100P inhibitors as chemotherapeutics for PC (159). In addition, through *in vitro* cell experiments and using mouse models, it has been found that cromolyn may bind to S100P to prevent activation of RAGE, thereby inhibiting S100P-mediated PC growth, survival and invasiveness (145). A new RAGE antagonist peptide (RAP) can inhibit the interaction of S100P and RAGE *in vivo* and *in vitro* at micromolar concentrations and can inhibit the growth and metastasis of PDAC (144). Metformin is closely related to the morbidity, mortality, proliferation, invasion and metastasis of a variety of tumors (160–162). Researchers cultured pancreatic cancer BxPC-3 and AsPC-1 cells *in vitro* and found that metformin can inhibit the growth of PC cells in a time- and dose-dependent manner. Metformin may inhibit the invasion and metastasis of PC cells by inhibiting the expression of the metastasis-related genes S100A4 and MMP-9. Wang et al. treated human pancreatic cancer Capan-1 cells with different concentrations of dexmedetomidine (Dex) and demonstrated for the first time that Dex inhibits the proliferation of PC cells and promotes cell apoptosis by upregulating the expression of miR-526b-3p and inhibiting the expression of S100A4 (163). Although there is currently no clinically effective drug for treatment of PC with S100 protein overexpression, various experimental results have provided a basis for the potential effectiveness of targeting S100 proteins to treat PC.

## Nomenclature

The S100 protein family is a significant potential biomarker for early PC diagnosis or prognosis determination, but this notion needs to be further supported by evidence obtained from large samples and multiple centers with different populations. Current studies mostly focus on certain S100 proteins or their combination with known clinical diagnostic markers as PC biomarkers, and whether S100 family proteins can be combined with each other to improve diagnostic performance is still unknown. An imbalance in S100 proteins plays a central role in PC progression and metastasis. Thus, therapies targeting S100 family members are expected to improve the survival and prognosis of PC patients. Although the biological characteristics and mechanisms of these proteins in PC are not yet clear, the current meaningful findings still highlight the important value of the S100 protein family in future research on PC.

## Author Contributions

YW conceived this review and drafted the manuscript. QZ and FG drew the figures. MC collected relevant references. XT and DD reviewed the manuscript. All authors contributed to the article and approved the submitted version.

## Conflict of Interest

The authors declare that the research was conducted in the absence of any commercial or financial relationships that could be construed as a potential conflict of interest

## Publisher’s Note

All claims expressed in this article are solely those of the authors and do not necessarily represent those of their affiliated organizations, or those of the publisher, the editors and the reviewers. Any product that may be evaluated in this article, or claim that may be made by its manufacturer, is not guaranteed or endorsed by the publisher.
